# Monkeypox Virus Infection in 18-Year-Old Woman after Sexual Intercourse, France, September 2022

**DOI:** 10.3201/eid2901.221643

**Published:** 2023-01

**Authors:** Alexandre Vallée, Audrey Chatelain, Marie Carbonnel, Catherine Racowsky, Erwan Fourn, David Zucman, Jean-Marc Ayoubi

**Affiliations:** Foch Hospital, Suresnes, France (A. Vallée, A. Chatelain, M. Carbonnel, C. Racowsky, E. Fourn, D. Zucman, J.-M. Ayoubi);; University of Versailles, Versailles, France (A. Chatelain, M. Carbonnel, J.-M. Ayoubi)

**Keywords:** Monkeypox, viruses, outbreak, women, genital, vaccine, prevention, sexually transmitted infections, France

## Abstract

A monkeypox virus outbreak has spread worldwide since April 2022. We report a young woman in France positive for monkeypox virus transmitted through oral and vaginal sex. Ulceronecrotic lesions developed intravaginally and around her vulva. Health professionals should become familiar with all aspects of infection from this virus, including possible vertical transmission.

A monkeypox virus (MPXV) outbreak has spread worldwide since April 2022. Although the risk to the general public was previously considered low, the World Health Organization is now responding to this outbreak as a high priority to avoid further spread (*1*). The community of men who have sex with men appears to be particularly exposed (*2*), although rare cases among women have been described. We report a sexually transmitted case of MPXV with typical genital lesions in a young woman in France. The study was approved by the Institutional Review Board of Foch Hospital (IRB no. IRB00012437 [approval no. 22-10-02]), Suresnes, France. Written consent for publication was obtained from the patient for clinical information and photographs. Deidentified data were securely transferred and stored.

On September 7, 2022, an 18-year-old woman sought care at the outpatient clinic of Foch Hospital for symptoms such as fever, myalgia, and multiple eruptions on her vulva that began 7 days earlier (Figure). She reported a headache began on September 2, 2022, followed by feverish episodes on September 2 that stopped on September 7. The first eruptions appeared on September 2 on the gluteal area and then spread. Rashes on her hands and wrists appeared on September 7. At examination, the patient showed no odynophagia, coughing, or sputum. A gynecological examination showed ulceronecrotic lesions around the vulva and intravaginally. The cutaneous lesions were infracentimetric pustules located on the torso, fingers, and palms of the hands and above the intergluteal groove. We observed bilateral laterocervical and inguinal lymphadenopathies, but no axillary lymphadenopathy or anal lesions were evident.

**Figure:**
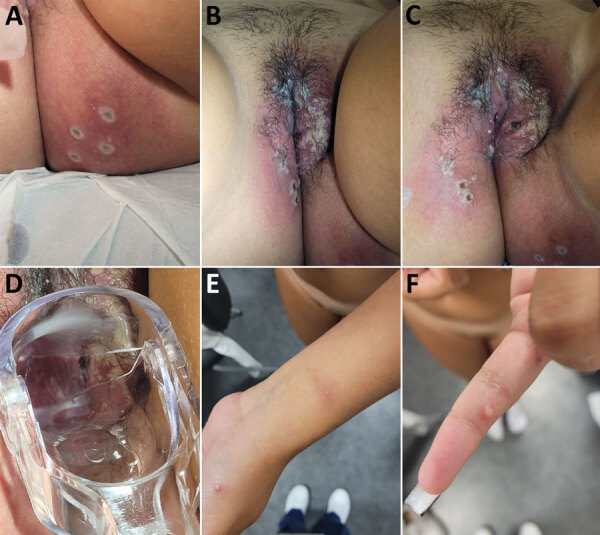
Pustules in gluteal area (A), genital area (B, C), intravaginal area (D), arm and hand (E), and finger (F) in young woman with monkeypox virus infection after sexual intercourse, France, September 2022.

Tests for chlamydia and gonococcus on pharyngeal and self-collected anal swabs were negative, and no previous sexually transmitted infections were reported for the patient or her boyfriend. Tests for HIV, syphilis, herpes, toxoplasmosis, rubella, and hepatitis (A, B, and C) were all negative for both persons. A real-time reverse transcription PCR, designed at the French National Reference Center of Orthopoxvirus, was performed on a pharyngeal swab sample from the patient on September 7 and tested positive for MPXV on September 8.

The patient had been in a relationship for 9 months with her boyfriend and had only had vaginal and oral intercourse with him; the most recent intercourse was 8 days before her first symptoms. On August 25, 2022, after a vacation in the southwest of France, her boyfriend began experiencing fever, pimples on his penis, and swollen inguinal glands. His rashes disappeared on September 1. We have no additional information for him. The main transmission hypothesis for MPXV in this case is oral and vaginal sex with the patient’s boyfriend. She was not hospitalized but placed in confinement in her home. On October 12, the patient was seen at Foch Hospital for follow up, at which time the rashes were beginning to disappear (Appendix).

Most current MPXV outbreaks in Europe have involved young men who have sex with men who attend festivals and other public events (*3*). Conversely, data regarding MPXV cases in women are sparse; only a few cases have been reported in the literature (*4*). To date, only a small proportion of infections have been reported in women; only 1.2% of total cases in Europe have been reported in women (*5*). Our patient had a regular sexual partner who had MPXV symptoms, thus reinforcing that sexual transmission might play a predominant role in the outbreak (*2*).

Pharyngitis and rectal symptoms are being increasingly described as occurring after traditional skin lesions in MPXV infections (*6*), although these symptoms were not evident in our patient. The most reported clinical symptoms are painful perianal and genital lesions, together with fever, lymphadenopathy, headache, and malaise. Classic cases of MPXV included a febrile prodrome followed by generalized rash, although the ongoing outbreak has been characterized mainly by painless anogenital lesions, often without a prodrome. However, because of observed variability in clinical manifestations, the spread of the current MPXV outbreak might be underestimated.

To date, only 10 cases in pregnancy have been reported worldwide, predominantly in Brazil and the United States (*7*). Those cases were mainly reported by local media, and none were severe. However, studies in pregnant women remain limited. A transplacental transmission to the fetus might be responsible for congenital MPXV, although no cases of fetal malformations or death have been reported. Nevertheless, cases of vertical transmission with clinical signs of MPXV infection, such as cutaneous maculopapular lesions on the head, trunk, and extremities and hydrops, have been reported (*8*). A neonate born from an infected mother in the United States received prophylactic vaccinia immunoglobin and did not develop MPXV disease. Of note, the ACAM2000 vaccine (Sanofi, https://www.sanofi.com) is contraindicated during pregnancy because it is a vaccinia virus (live virus); other vaccines, such as third-generation vaccines (LC16m8 or JYNNEOS [Bavarian Nordic, https://www.bavarian-nordic.com]), are preferred in pregnant women (*9*). It is imperative that health professionals become familiar with all aspects of this disease affecting women (*10*).

Appendix Additional information about monkeypox virus infection in 18-year-old woman after sexual intercourse, France, September 2022
