# Supplementary material for: Monkeypox Virus Infection in 18-Year-Old Woman after Sexual Intercourse, France, September 2022
Source: Emerg Infect Dis. 2023 Jan;29(1):219–22. doi: 10.3201/eid2901.221643 (PMC9796185; doi:10.3201/eid2901.221643)
Supplement: Appendix — Additional information about monkeypox virus infection in 18-year-old woman after sexual intercourse, France, September 2022 [file 22-1643-Techapp-s1.pdf]

Article DOI: https://doi.org/10.3201/eid2901.221643

Monkeypox Virus Infection in 18-Year-Old Woman after Sexual Intercourse, France, September 2022

Appendix

Timeline of Monkeypox Virus Infection

August 25, 2022: Boyfriend develops fever and rashes.

August 26, 2022: Oral and vaginal intercourse between the woman and her boyfriend

September 1, 2022: Rashes disappear in boyfriend (no additional information available).

September 2, 2022: Feverish episode for the female

September 2, 2022: First eruptions on gluteal area for woman (photographed on September 7, 2022) (Appendix Figure 1)

September 3, 2022: First eruptions on genital area (photographed on September 7, 2022) (Appendix Figure 2)

September 7, 2022: Clinical visit at Foch Hospital, Suresnes, France. Rashes on hands and wrists of the woman have developed (photographed on September 7, 2022) (Appendix Figure 3)

September 7, 2022: Intravaginal lesions (photographed on September 7, 2022) (Appendix Figure 4)

September 8, 2022: Woman tests positive for monkeypox virus by reverse transcription PCR positive

October 12, 2022: Second visit at Foch Hospital. Beginning of disappearance of rashes for woman (photographed on October 12, 2022) (Appendix Figure 5)

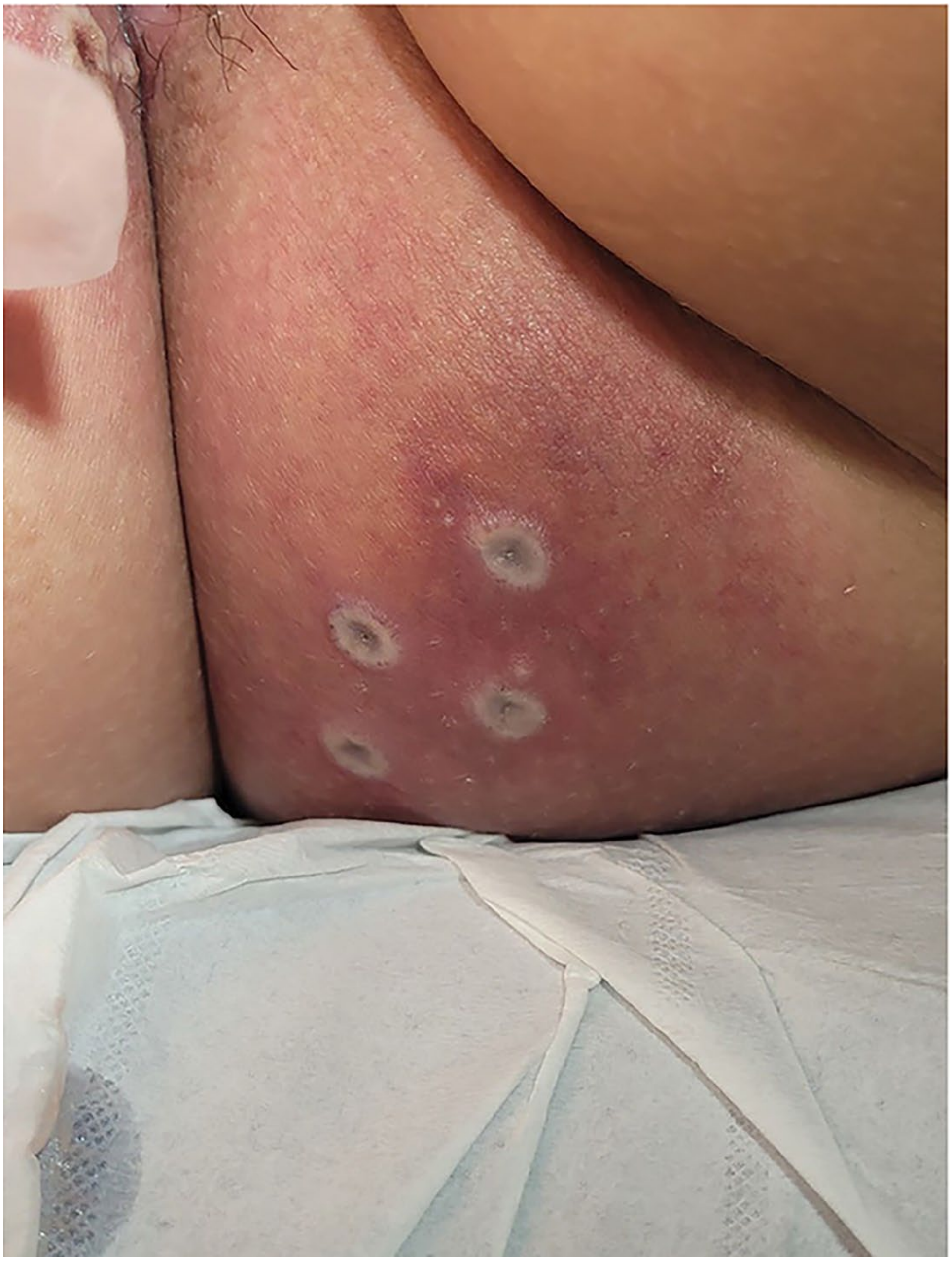

Appendix Figure 1. Pustules in gluteal area of young woman with monkeypox virus infection after sexual intercourse, France, September 2022

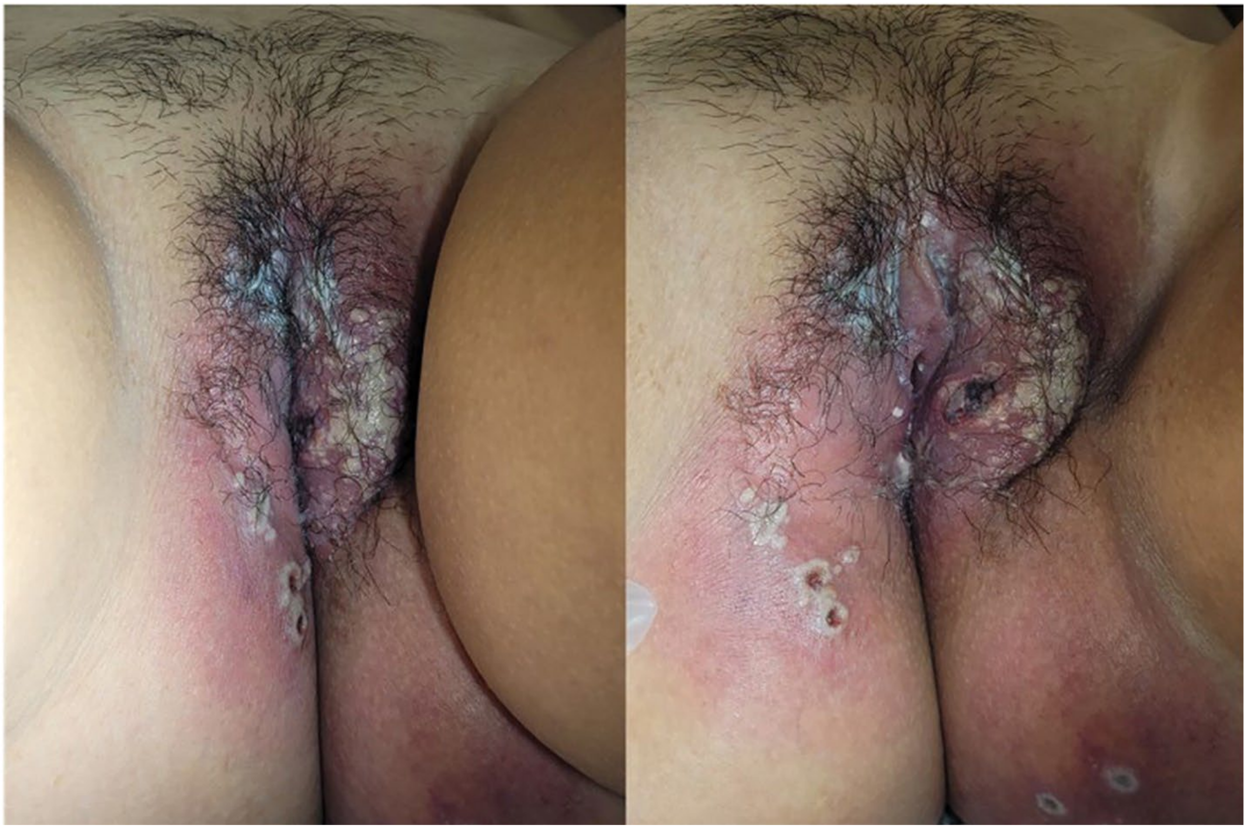

Appendix Figure 2. Pustules in genital area of young woman with monkeypox virus infection after sexual intercourse, France, September 2022

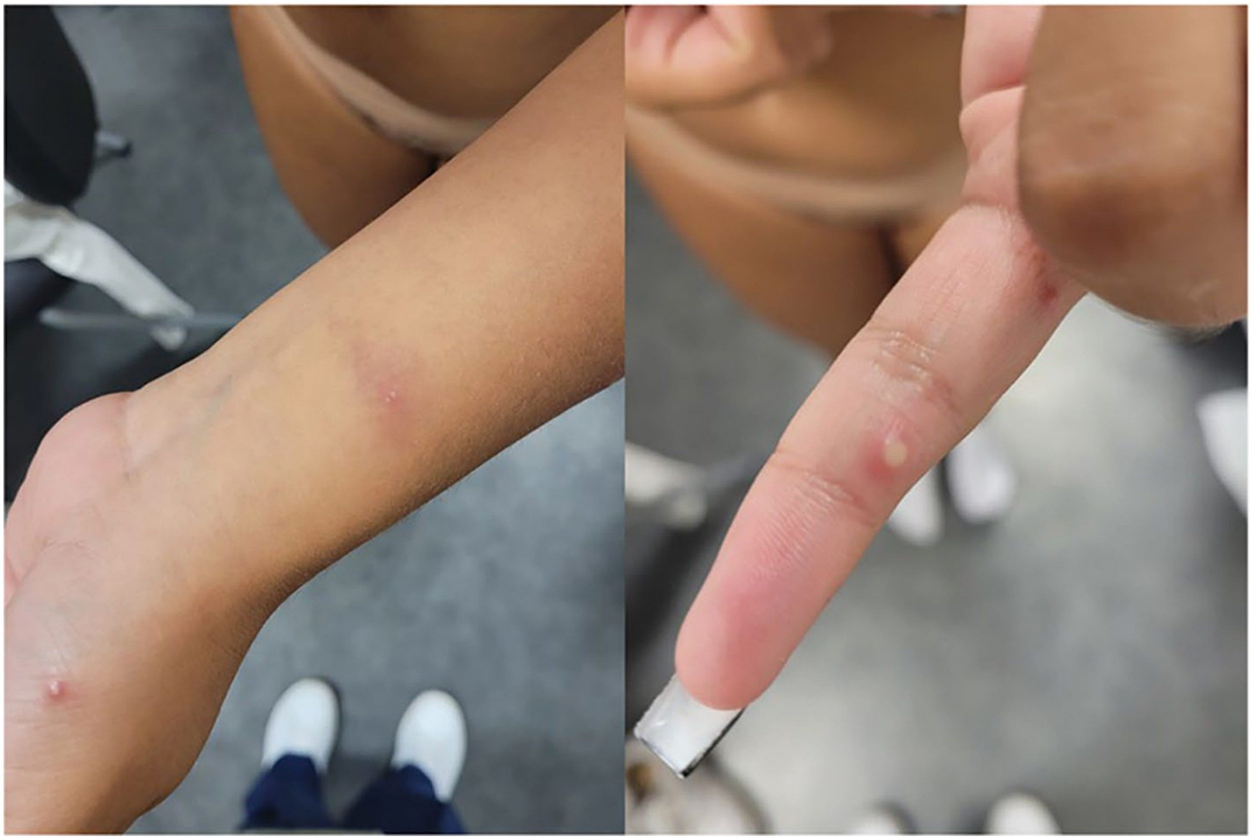

Appendix Figure 3. Pustules on arm, hand, and finger of young woman with monkeypox virus infection after sexual intercourse, France, September 2022

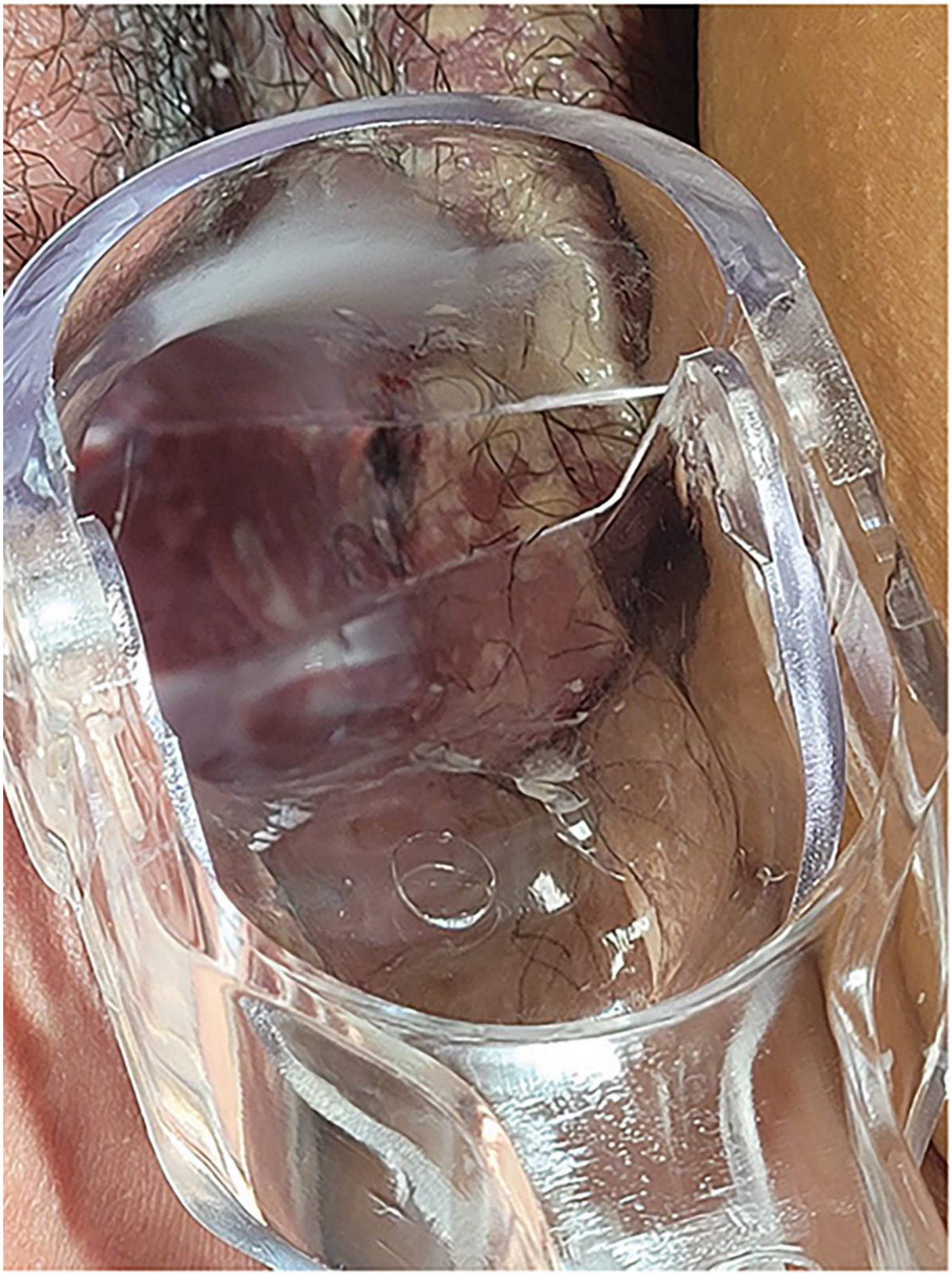

Appendix Figure 4. Pustules in intravaginal area of young woman with monkeypox virus infection after sexual intercourse, France, September 2022

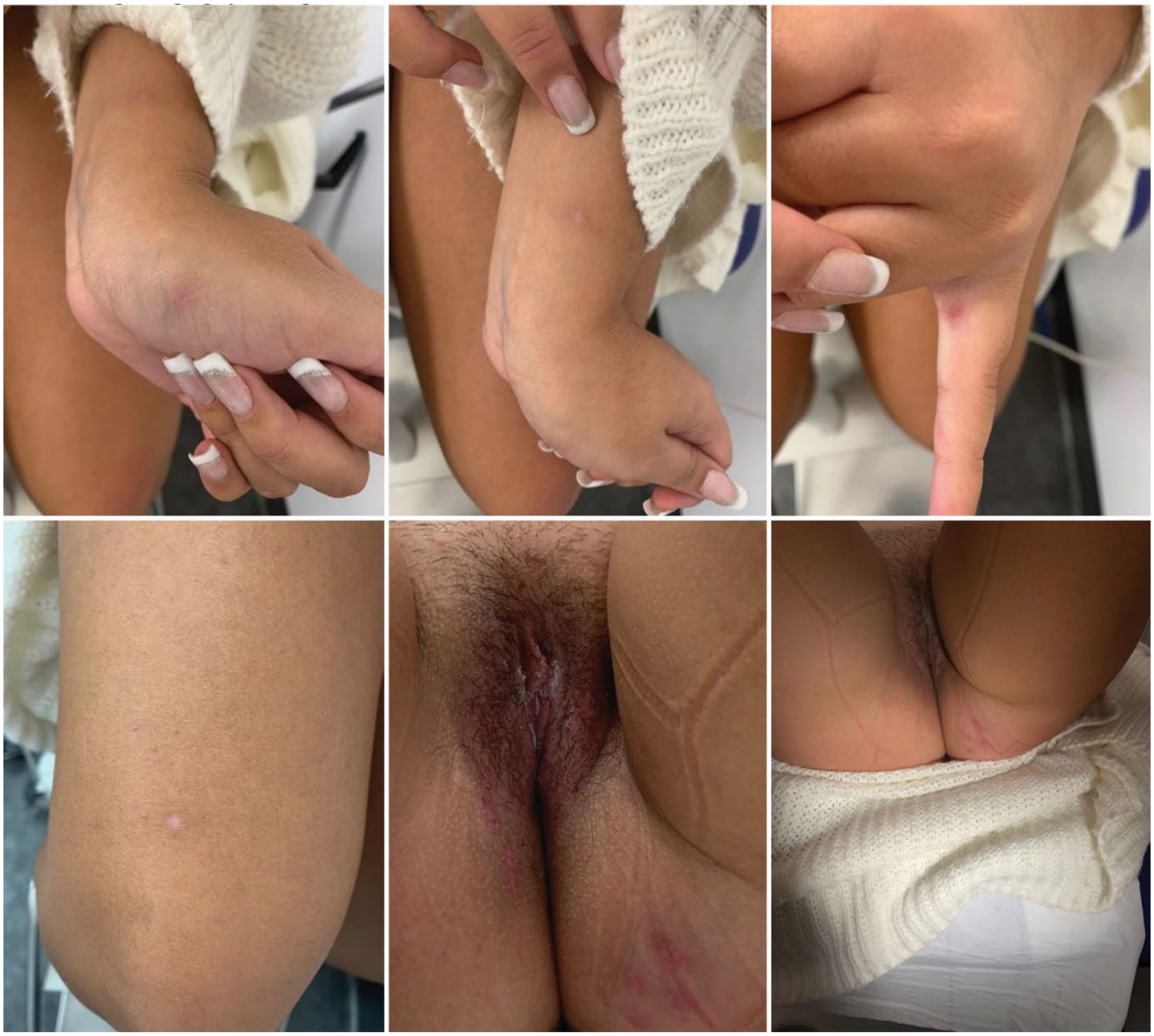

Appendix Figure 5. Healing pustules in young woman with monkeypox virus infection after sexual intercourse, France, September 2022
